# Regulation of Cr(VI)-Induced Premature Senescence in L02 Hepatocytes by ROS-Ca^2+^-NF-*κ*B Signaling

**DOI:** 10.1155/2022/7295224

**Published:** 2022-02-18

**Authors:** Yujing Zhang, Gang Yang, Shuai Huang, Xinyue Yang, Fengyan Yuan, Yinghui Song, Sulai Liu, Xing Yu

**Affiliations:** ^1^Key Laboratory of Molecular Epidemiology of Hunan Province, School of Medicine, Hunan Normal University, Changsha, China; ^2^Department of Hepatobiliary Surgery, Hunan Provincial People's Hospital/The First Affiliated Hospital of Hunan Normal University, Changsha 410015, China; ^3^Key Laboratory of Model Animals and Stem Cell Biology of Hunan Province, School of Medicine, Hunan Normal University, Changsha, China; ^4^Institute for Glycomics, Griffith University, Gold Coast, Queensland, Australia

## Abstract

Stress-induced premature senescence may be involved in the pathogeneses of acute liver injury. Hexavalent chromium [Cr(VI)], a common environmental pollutant related to liver injury, likely leads to premature senescence in L02 hepatocytes. However, the underlying mechanisms regarding hepatocyte premature senility in Cr(VI) exposure remain poorly understood. In this study, we found that chronic exposure of L02 hepatocytes to Cr(VI) led to premature senescence characterized by increased *β*-galactosidase activity, senescence-associated heterochromatin foci, G1 phase arrest, and decreased cell proliferation. Additionally, Cr(VI)-induced senescent L02 hepatocytes showed upregulated inflammation-related factors, such as IL-6 and fibroblast growth factor 23 (FGF23), which also exhibited reactive oxygen species (ROS) accumulation derived from mitochondria accompanied with increased concentration of intracellular calcium ions (Ca^2+^) and activity of nuclear factor kappa B (NF-*κ*B). Of note is that ROS inhibition by N-acetyl-Lcysteine pretreatment not only alleviated Cr(VI)-induced premature senescence but also reduced the elevated intracellular Ca^2+^, activated NF-*κ*B, and secretion of IL-6/FGF23. Intriguingly, the toxic effect of Cr(VI) upon premature senescence of L02 hepatocytes and increased levels of IL-6/FGF23 could be partially reversed by the intracellular Ca^2+^ chelator BAPTA-AM pretreatment. Furthermore, by utilizing the NF-*κ*B inhibitor pyrrolidine dithiocarbamate (PDTC), we confirmed that NF-*κ*B mediated IL-6/FGF23 to regulate the Cr(VI)-induced L02 hepatocyte premature senescence, whilst the concentration of intracellular Ca^2+^ was not influenced by PDTC. To the best of our knowledge, our data reports for the first time the role of ROS-Ca^2+^-NF-*κ*B signaling pathway in Cr(VI)-induced premature senescence. Our results collectively shed light on further exploration of innovative intervention strategies and treatment targeting Cr(VI)-induced chronic liver damage related to premature senescence.

## 1. Introduction

Cellular senescence, originally discovered by Hayflick and Moorhead, has been regarded as a permanent state with cell cycle arrest which is implicated in a number of pathological conditions such as age-related diseases [1]. Compelling evidence in recent years has shown that accumulation of senescent cells is the main feature of aging amongst various species [2, 3]. It is believed that cell senescence with the ability to survive rather than divide could be induced by a wide variety of factors, including the cell division limit (termed replicative senescence) and various damaging stimuli (termed stress-induced premature senescence) [3, 4]. Stress-induced premature senescence causes abnormal accumulation of senescent-like cells to damage cell function and tissue repair. Studies have provided vital evidence in the close correlation between premature senescence and liver diseases. Cellular senescence of hepatocytes, hepatic stellate cells, or hepatic sinusoidal endothelial cells have been observed in liver fibrosis [5]. Moreover, some disease paradigms indicated that cirrhosis in Werner's syndrome was a sign of premature senescence [6]. Hence, elucidation of the underlying mechanisms for cell premature senility may be a key to our further understanding of liver disease pathogenesis.

There are some markers to identify senescent cells in addition to cell cycle arrest, including the increased p16 and p21 gene expression, enhanced activity of senescence-associated *β*-galactosidase (SA-*β*-gal), and senescence-associated secretory phenotype (SASP) referring to inflammatory factors and other relevant cytokines. It is well documented that the occurrence of senescence primarily attributed to the secretion of inflammatory molecules that influence the function of adjacent cells. Many inflammatory cytokines, such as IL-6 and IL-8, are closely related to premature senescence induced by stimuli [7]. Further, fibroblast growth factor (FGF) 23, a proinflammatory circulating hormone, has also been stated as a potent phosphaturic factor associated with premature senescence. Inflammation is generally defined as the response to both endogenous and exogenous stimuli, including damaged cells or oxidative stress [8]. Moreover, some theories postulated that high levels of reactive oxygen species (ROS) were markedly associated with aging rate. For example, during aging, oxidative stress-induced mitochondrial DNA mutations caused production of ROS aggravating mitochondrial dysfunction, which then led to elevated liver ballooning degeneration [9]. We also demonstrated in our early study that accumulated ROS was considerably correlated with the premature senescence of L02 hepatocytes [10]. These results imply that oxidative stress drives cell premature aging to aggravate liver injury. Further, there is a great deal of research which has provided critical insights into the role of ROS in the toxicity of hexavalent chromium [Cr(VI)] which could be commonly found in the environment.

Cr(VI), classified as a human carcinogen evidenced by the increased lung cancer incidence amongst workers via the inhalation route of exposure, is frequently exposed in multiple occupational environments on account of its application in plating, welding, and leather tanning. Recently, oral exposure to Cr(VI) is of increasing concern as the water pollution caused by Cr(VI) has become a serious public problem. After cell entry, Cr(VI) is reduced to trivalent chrome [Cr(III)] that played key roles in the toxicity, accompanying with the formation of intracellular ROS [11]. Epidemiological and experimental evidence have indicated that oral exposure to Cr(VI) via water consumption may lead to increased incidences of liver damage [12]. It has to be noted that the main focus so far is on the regulatory mechanisms of acute hepatotoxicity such as cell cycle arrest, cell apoptosis, and autophagy, but the significance of the chronic hepatotoxicity induced by Cr(VI), especially premature senescence or liver cancers, is somewhat less attended. In this study, we aim to further investigate the molecular mechanisms of premature senescence sensitized by chronic low-dose Cr(VI) exposure in hepatocytes.

Ca^2+^ is recognized as the significant second messenger that participates in various aspects of cellular functions including protein secretion, gene transcription, and cell growth in a wide variety of cells [13, 14]. Intracellular Ca^2+^ is primarily stored in the endoplasmic reticulum (ER) or mitochondria, while the delicate regulation of Ca^2+^ homeostasis requires the participation of plasma membranes, organellar Ca^2+^ channels, exchangers, and transporters. As a result, any disturbance of Ca^2+^ homeostasis may give rise to cell damage, for instance, apoptosis or necrosis. We previously reported that acute Cr(VI) exposure could cause cell apoptosis of hepatocytes via intracellular Ca^2+^ overload [15]. Alternative to death, certain stress factors may induce cells to develop cellular senescence. It has been shown in our early data that 10 nM Cr(VI) exposure to L02 hepatocytes for 24 h twice a week for 4 consecutive weeks could induce premature cellular senescence [10]. Some studies also implicated the roles of Ca^2+^ homeostasis in senescence progression [16]. For instance, aged rat neurons exhibit elevated Ca^2+^ levels, increased Ca^2+^ release from the ER, enhanced Ca^2+^ flux between ER and mitochondria, and changed expression of key proteins regulating cellular Ca^2+^ homeostasis [17]. Furthermore, cytoplasmic Ca^2+^ overload is closely related to the senescence of human endothelial cells and atrial myocytes [18, 19]. Therefore, we aimed to investigate the involvement of Ca^2+^ in Cr(VI)-induced premature senescence of L02 hepatocytes as well as the related mechanism.

Nuclear factor-*κ*B (NF-*κ*B) is well known as an ubiquitous transcription factor in various cells, which is along with inflammation and Ca^2+^ homeostasis to regulate cell survival and death [20]. p65 (RelA), c-Rel, RelB, NF-*κ*B1 (p105/p50), and NF-*κ*B2 (p100/p52) belong to the NF-*κ*B family, with the most common form being the p65/p50 dimer. Typically, NF-*κ*B is present in the cytoplasm as an inactive form in complex with inhibitors of *κ*B (I*κ*B) proteins. When stimulated by canonical (classical) and noncanonical pathways, NF-*κ*B will be rapidly translocated into the nucleus to regulate the expression of target genes [21], but silent information regulator 1 (Sirt1) could reduce the transcriptional activity of NF-*κ*B via regulating its deacetylation. Interestingly, a study has shown that Cr(VI) exposure lead to inflammation response in rat liver via inhibiting the deacetylation of Sirt1 resulting in the increased level of acetylated NF-*κ*B-p65 and upregulated proinflammatory factors IL-1*β* and TNF-*α* [22]. Indeed, recent reports have demonstrated a key physiological role for the NF-ĸB signaling pathway in humans and rodents upon aging and cellular senescence [23–25]. In addition, activation of NF-*κ*B is closely relevant to some aging-related chronic diseases, such as Alzheimer's disease or Parkinson's disease [26]. However, there continues to be an untapped potential for finer-grained effects of NF-*κ*B signaling on premature senescence induced by Cr(VI) that would demand further investigation.

The aim of this study is to further explore the underlying mechanisms of premature senescence induced by chronic Cr(VI) exposure. Our results indicated that, upon intracellular Ca^2+^ overload mediated by accumulation of ROS, markers of Cr(VI) induced premature senescence, such as IL-6 and FGF-23, were modulated by the NF-ĸB signaling pathway. Our data will provide further insights into molecular profiles of Cr(VI)-induced premature senescence of hepatocytes and may contribute to the development of novel therapeutic strategies in fight against age-related diseases.

## 2. Materials and Methods

### 2.1. Materials

Potassium dichromate (K_2_Cr_2_O_7_) was purchased from Sigma-Aldrich (St. Louis, USA). Antibodies against p53(A3185), Phospho-p53-S15 (AP0083), TriMethyl-Histone H3-K9 (A2360), and Murine Double Minute 2 (MDM2, A0345) were obtained from ABclonal Technology (Wuhan, China). Antibodies against p21Waf1/Cip1 (#2947), PCNA (#13110), and Phospho-NF-*κ*B p65 (Ser536) (#3033) were purchased from Cell Signaling Technology (Danvers, MA, USA). Antibodies against NF-*κ*B p65 (66535-1-Ig), IKK*αα* (40905), and I*κ*B (40903) were purchased from Proteintech (Wuhan, China). The ROS inhibitor *N*-acetyl-Lcysteine (NAC) and the NF-*κ*B inhibitor pyrrolidine dithiocarbamate (PDTC) were purchased from Beyotime Technology (Shanghai, China). The intracellular calcium chelator BAPTA-AM was purchased from UE EVERBRIGHT®INC. (Wuhan, China).

### 2.2. Cell Culture and Treatment

L02 hepatocytes, a gift from Nanjing Medical University, were cultured in RPMI-1640 medium (Gibco, Grand Island, USA) supplemented with 10% foetal bovine serum (FBS, Gibco, Grand Island, USA) and 1% penicillin-streptomycin at 37°C and 5% CO2. In the present study, the cells were treated with PBS or 10 nM Cr(VI) twice a week for 24 h for 4 consecutive weeks.

### 2.3. Senescence-Associated *β*-Galactosidase (SA-*β*-Gal) Activity Assay

*β*-Galactosidase Staining Kit (Beyotime Technology, Wuhan, China) was used to detect the SA-*β*-gal activity according to the manufacturer's instruction. In brief, cells were seeded in six-well tissue culture plates at a concentration of 2 × 105 cells/well. At the end of incubation for 6~8 h, the cells were fixed by 4% paraformaldehyde for 15 min at room temperature and washed with PBS and then incubated with the staining solution at 37°C for 12~14 h. After washed with PBS, cells were then observed using microscopy (Leica, Weztlar, German). In order to evaluate the percentage of senescent cells, L02 cells were stained with SA-*β*-gal and then processed to determine the percentage of the senescent cells by counting positively stained cells under the microscope. The percentage of premature senescent cells was measured using the following equation: percentage of premature senescent cells = (positively stained cells/total cells) × 100%. The cells were counted manually instead of using the automated counting instrument.

### 2.4. Senescence-Associated Heterochromatin Foci (SAHF) Assay

Cell nuclei were stained by DAPI to determine the morphological evidence of premature cellular senescence. After incubation overnight, cells seeded in six-well tissue culture plates were fixed with 4% paraformaldehyde for 20 min at room temperature and washed with PBS for three times. Then, DAPI (1 mg/ml) was used to stain the fixed cells for 20 min. After washed with PBS, cells were observed by fluorescent microscopy with a peak excitation wavelength at 340 nm. The fluorescent intensity was quantified by ImageJ software.

### 2.5. Cell Cycle Analysis

The L02 and premature senescent L02 hepatocytes were seeded into 60 mm plates at 4.5 × 105 cells/wells and were placed in an incubator overnight. Flow cytometry analysis was performed to detect the cell cycle distribution when the cells were at 80~90% confluence. Cells were collected and washed with PBS before fixed in 75% cold ethanol for 2~4 h at 4°C. Then, the cells were washed once with PBS and resuspended in 200 *μ*l PBS with RNase and PI for 30 min in the dark at 37°C. The samples were immediately assayed by a FACS BD Aria III flow cytometer.

### 2.6. EdU Staining

Click-iT EdU Imaging Kit (UE EVERBRIGHT®INC., Wuhan, China) was used to detect the EdU staining following the manufacturer's protocol. In brief, the cells were incubated with EdU working fluid for 4 h after treatment, fixed with 4% paraformaldehyde for 20 min, and incubated with 5 mg/ml glycine for 5 min. After washing twice with 3% bovine serum albumin (BSA) in PBS, the cells were permeabilized with 0.5% Triton X-100 in PBS for 20 min. The cells were again washed with 3% BSA in PBS for three times and then stained with a Click-iT reaction cocktail containing for 30 min away from light. The cells were washed further with 3% BSA in PBS and washed with PBS for the following DNA staining. Subsequently, the cells were incubated with Hoechst 33342 (5 *μ*g/ml) for 30 min. The cells were observed by fluorescent microscopy after washed with PBS for three times. All procedures were conducted at room temperature. The fluorescent intensity was quantified by ImageJ software.

### 2.7. Western Blot

For Western blot, 2 × 105 cells were seeded in six-well tissue culture plates, exposed to the indicated treatment, then were scraped and lysed with RIPA lysis buffer (Beyotime Technology, Shanghai, China) or the nuclear and cytoplasmic protein extraction kit (P0027, Beyotime Technology, Shanghai, China) containing protease and phosphatase inhibitors (Pierce Protease and Phosphatase Inhibitor Mini Tables). After running in the 5–15% SDS-PAGE gels and transferred onto NC membrane (Life Technologies), membranes were blocked for 60 min at room temperature with 5% nonfat milk in TBS with 0.1% Tween 20 and incubated overnight at 4°C with the primary antibodies. The primary antibodies incubated with horseradish peroxidase-conjugated secondary antibodies (ABclonal Technology, Wuhan, China) for 1 h at room temperature and immunoreactive bands were detected using an ECL kit. The density of the immunoreactive bands was analyzed using ImageJ software.

### 2.8. ELISA

The cells seeded in the 60 mm tissue culture plates; culture supernatant was collected and kept at -80°C. ELISA was used to detect the concentration of IL-6 (Dingguo Technology, Beijing, China) and FGF23 (Abcam, Cambridge, England) in the culture supernatant following the manufacturer's instructions.

### 2.9. Intracellular ROS Measurement

DCFH-DA fluorescence probe (Beytime, Wuhan, China) was used to assay the accumulation of intracellular ROS. Briefly, cells (2 × 105 cells/well) were seeded in the six-well tissue culture plate. After determined treatment, the treated cells were washed twice with PBS and incubated with 10 *μ*M DCFH-DA in RPMI1640 medium without FBS for 40 min at 37°C in the dark, and the stained cells were then analyzed using a FACS BD Aria III flow cytometer or fluorescent microscopy.

### 2.10. Mitochondrial Membrane Potential (MMP, ΔΨ*m*) Detection

Tetramethylrhodamine methyl ester (TMRM, Thermo Fisher Scientific) and JC-1 (UE EVERBRIGHT®INC., Wuhan, China) were used to detect the changes in Δ*ψm*. After treatment, the cells were seeded in the 96-well plate and loaded TMRM, and the mitochondria was located using Mito-tracker Green (Beyotime Technology, Shanghai, China) that was not influenced by MMP and JC-1 following introduction, then the fluorescent microscopy and spectrofluorometry were carried out immediately.

### 2.11. ATP Content Analysis

Cells were seeded into 60 mm plates at the density of 5 × 105 cells/well before lysed with lysis buffer. The supernatant was collected after centrifugation, and 100 *μ*l ATP detection solution was added to a nontransparent 96-well plate at RT for 5 min. ATP standard reagent or cell supernatant was added to each well and mixed thoroughly. Then, the spectrofluorometry was used for ATP content detection.

### 2.12. Mitochondria Mass Detection

Cells were inoculated into a nontransparent 96-well plate at a density of 8 × 10^3^ cells/well. NAO was added to each well (5 *μ*M) for 30 min in the dark. The spectrofluorometry (excited wavelength 485 nm, emitted wavelength 530 nm) was used to detect the absorbance before the cells were washed by RPMI 1640 medium.

### 2.13. Measurement of mtDNA Copy Number

The mtDNA copy number was assayed by Real-Time PCR Detection System (ABI7300 plus) with the SYBR Green I detection method as the instruction. The mtDNA primer was designed to detect COXI: 5′-CAAACCTACGCCAAAATCCA-3′ and 5′-GAAATGAATGAGCCTACAGA-3′ [27]; the ACTB primers were 5′-CACCAGGGCGTGATGGT-3′ and 5′-CTCAAACATGATCTG GGTCAT-3′. The RT-PCR reaction is under the following conditions: 40 cycles of 95°C for 30 s, 60°C for 30 s; and 95°C for 5 s.

### 2.14. Mitochondrial Morphology Observation

The cells were seeded into 24-weel plate for 8~12 h and washed by PBS for 3 times. Then, the cells were loaded with Mitotracker Red CMX ROS (2 nM in FBS-free RPMI 1640 medium) for 5 min at 37°C in the dark. After washed by PBS, the cell nucleus was stained by Hoechst 3342. The cells were examined by a fluorescence microscope.

### 2.15. Intracellular and Mitochondrial Ca^2+^ Measurement

Intracellular Ca^2+^ concentration was evaluated as the fluorescence intensity of Fluo-3/AM determined by flow cytometry or spectrofluorometry. Briefly, the hepatocytes were incubated with 0.5 *μ*M Furo-3/AM for 60 min at 37°C in the dark and incubated for another 30 min after washed twice with PBS. The cells were then analyzed using a FACS BD Aria III flow cytometer or fluorescent microscopy.

For mitochondrial Ca^2+^ measurement, cells probed with Rhod-2 were placed in a quartz cuvette, and fluorescence was evaluated by a F7000 HITACHI spectrofluorometer. Samples were excited at 550 nm, and fluorescence was measured at 590 nm. Mitochondrial Ca^2+^ concentration was measured as fluorescence intensity in relation to the initial fluorescence intensity.

### 2.16. Statistical Analysis

Statistics were analyzed using the SPSS 19.0. All quantitative data are presented as the mean ± SD from 3 independent experiments. Multiple group comparisons were performed using ANOVA, while statistical comparisons between two groups were made using Student's *t*-test. *p* < 0.05 was considered significant.

## 3. Results

### 3.1. Cr(VI)-Induced Premature Cellular Senescence of L02 Hepatocyte

**Figure 1:**
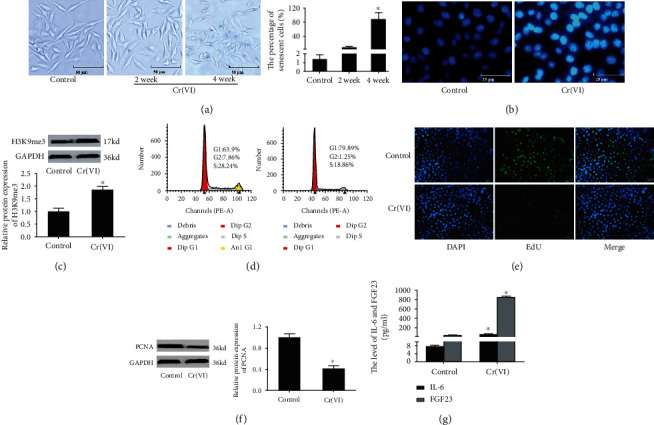
Cr(VI)-induced premature senescence in L02 hepatocytes. (a) The L02 hepatocytes were treated with 10 nM Cr(VI) or PBS for 24 h twice a week for 2 or 4 weeks. The activity of SA-*β*-gal was assayed by *β*-Galactosidase Staining Kit (200x), and the percentage of senescent cells was showed in bar graph. After the L02 hepatocytes treated with Cr(VI) for 4 weeks, (b) the SAHF was examined by DAPI staining (400x), and (c) the protein level of H3K9me3 was determined by Western blot. (d) The cell cycle distribution was analyzed by flow cytometry analysis. (e) Edu was used to detect the inhibition of proliferation of L02 hepatocytes with Cr(VI) exposure (200x). (f) The expression of PCNA protein was assayed by Western blot. (g)The secretion of IL-6 and FGF23 was examined by ELISA kit. ImageJ software was used to analyze the relative levels of proteins normalized to the expression of GAPDH. All experiments were repeated at least 3 times and expressed as mean ± SD. ^∗^*p* < 0.05, compared with control. For the sake of clarity, the same control GAPDH was applied to compare with all experimentally relevant proteins with the same exposure time detected on the same SDS-PAGE gel unless otherwise stated.

Previous research indicated that Cr(VI) was one of the main environmental factors for acute liver damage, especially cell apoptosis in hepatocytes [15]. To investigate the toxic effects of hepatocytes by chronic Cr(VI) exposure with a low dose, we were committed to undertake following experiments. The L02 hepatocytes were treated with 10 nM Cr(VI) or PBS for 24 h twice a week. We collected cells exposed to Cr(VI) for 0, 2, and 4 weeks and examined the activity of SA-*β*-gal. As shown in Figure 1(a), we found that the activity of SA-*β*-gal in the cells after Cr(VI) exposure for 4 weeks was statistically increased as compared to the control group, while there was no significant change of SA-*β*-gal in the cells after Cr(VI) exposure for 2 weeks. Next, we detected other biomarkers of senescent L02 cells with Cr(VI) exposure for 4 weeks. SAHF, usually regarded as the contributor to repress expression of proliferation-promoting genes, refers to bright punctate DNA foci when the cells were stained with DAPI.  Figure 1(b) showed that L02 hepatocytes exposed to Cr(VI) also resulted in an increase of SAHF, accompanying with the increased protein expression of H3K9me3, the marker of SAHF (Figure 1(c)). A state of permanent cell cycle arrest is the representative hallmark of senescence; the results in Figure 1(d) showed that the senescent L02 hepatocytes get arrested in G1 phase, while there was no obvious cell apoptosis in the both groups (Supplementary Figure S1). Additionally, the proliferation of L02 hepatocytes exposed to Cr(VI) was also tested by EdU staining. The result in Figure 1(e) validated that the proliferation of L02 cells was decreased in response to Cr(VI). Meanwhile, Western blot was applied to detect the levels of PCNA, which was the key regulator of cell proliferation. As seen in Figure 1(e), we observed a decreased protein expression of PCNA (Figure 1(f)). Besides, the protein levels of age-related biomarkers were significantly changed (Supplementary Figure S2). The supernatant of culture medium examined the levels of IL-6 and FGF23 using ELISA kits and found the increased levels of those two in the senescent cells (Figure 1(g)) Collectively, our results suggested that low-dose Cr(VI) exposure to L02 hepatocytes for 4 weeks could inhibit proliferation and induce premature cellular senescence.

### 3.2. ROS Derived from Mitochondria Is Involved in the Premature Senescence of L02 Hepatocytes Induced by Cr(VI)

**Figure 2:**
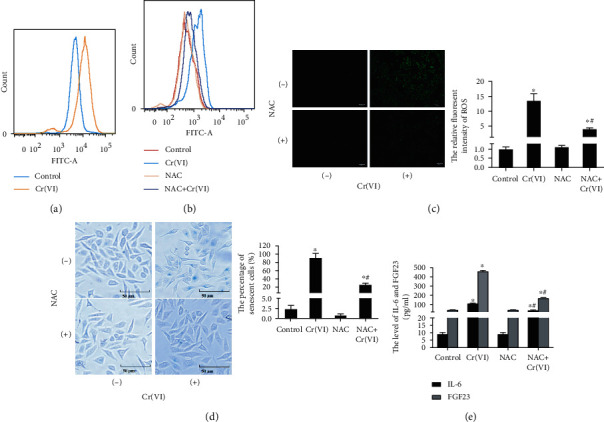
Cr(VI)-induced premature senescence via intracellular ROS formation. (a) The L02 cells were treated with Cr(VI) for 4 weeks and loaded with DCFH-DA. The mean DCF fluorescence was measured by flow cytometry. The cells were incubated for 1 h in the presence or absence of NAC (10 mM) prior to Cr(VI) exposure. The mean DCF fluorescent intensity was assayed by (b) flow cytometry and (c) fluorescent microscopy (200x), and ImageJ software was used to analyze the relative fluorescent intensity showed in bar graph. (d) After same treatment, the SA-*β*-gal activity was determined by *β*-Galactosidase Staining Kit (200x), and the percentage of senescent cells was showed in bar graph. (e) ELISA was used to detected the levels of IL-6 and FGF23. All experiments were repeated at least 3 times and expressed as mean ± SD. ^∗^*p* < 0.05, compared with control.

**Figure 3:**
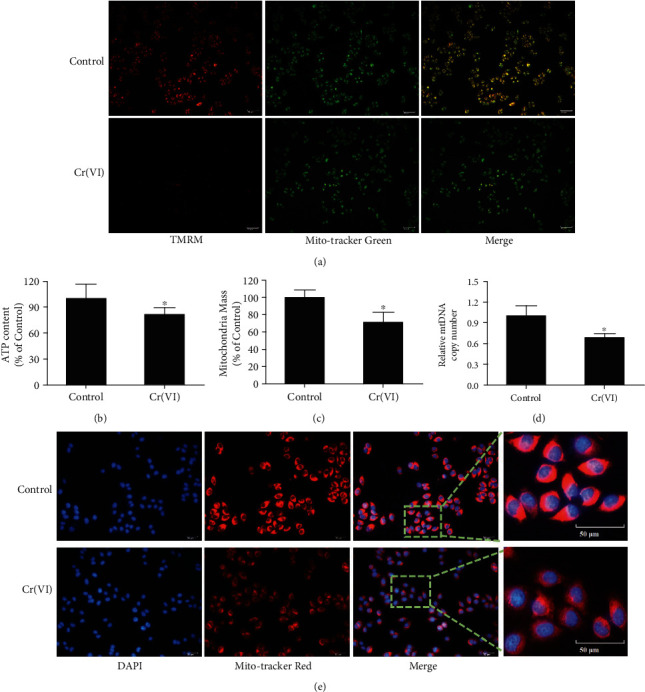
Low-dose and long-term expression of Cr(VI)-induced mitochondria dysfunction. After L02 cells were treated with Cr(VI) for 4 weeks, (a) the TMRM (red) staining assay for the detection of ΔΨ*m*, and Mito-Tracker Green (green) was used to locate the mitochondria (200x). (b) ATP content, (c) mitochondria mass, and (d) mtDNA copy number were also detected. (e) Mitochondrial morphology was determined by specific fluorescence probe Mito-Tracker Red CMXRos (red) (200x). All experiments were repeated at least 3 times and expressed as mean ± SD. ^∗^*p* < 0.05, compared with control; ^#^*p* < 0.05, compared with Cr(VI)-exposed group.

**Figure 4:**
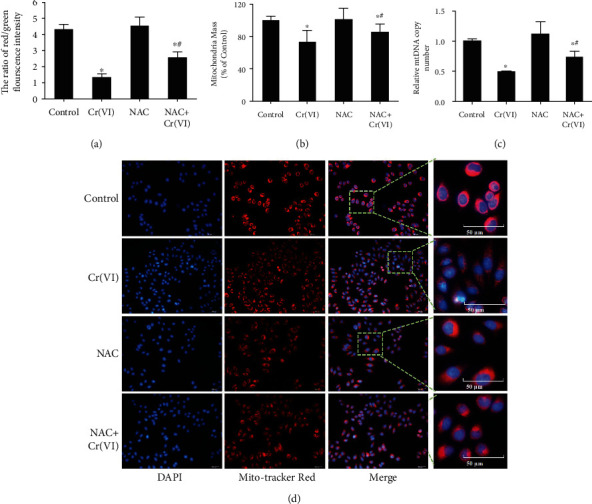
The specific antioxidant NAC reduced low-dose Cr(VI)-induced mitochondria dysfunction. (a) The NAC was used before Cr(VI) exposure in L02 hepatocytes, (a) then ΔΨ*m* was assayed by JC-1 using spectrofluorometry. (b) Mitochondria mass was assayed by NAO kit, and (c) mtDNA copy number was analyzed by RT-PCR. (d) Mitochondrial morphology was determined by Mito-Tracker Red CMXRos (red) (200x). All experiments were repeated at least 3 times and expressed as mean ± SD. ^∗^*p* < 0.05, compared with control; ^#^*p* < 0.05, compared with Cr(VI)-exposed group.

Excessive ROS accumulation has been viewed as an etiological factor related to cellular senescence [28]. In order to explore the role of ROS in premature senescence of L02 hepatocytes caused by Cr(VI), the fluorescent probe DCFH-DA was utilized to examine the levels of intracellular ROS. The results in Figure 2(a) demonstrated an increased fluorescence intensity in senescent L02 cells compared with the control. Subsequently, we used the antioxidant N-acetylcysteine (NAC) to pretreat L02 cells for 1 h prior to Cr(VI) exposure. Figures 2(b) and 2(c) revealed that accumulation of ROS was significantly inhibited by NAC, suggesting the inhibitory effects of NAC on ROS sensitized by Cr(VI). We also found in Figure 2(d) that pretreatment with NAC partially reduced the percentage of senescent cells induced by Cr(VI), which was in line with the result of SA-*β*-gal staining. Notably, the release of IL-6 and FGF23 was also diminished (Figure 2(e)). As for mounting evidence clearly shows that mitochondria are the key sources of ROS in cells, we thus explored whether ROS formation participated in premature senescence induced by Cr(VI) is related to mitochondrial dysfunction. TMRM was used to detect the ΔΨ*m* as the lipophilic cation accumulation in mitochondria in a manner dependent on ΔΨ*m*. As expected, the results in Figure 3(a) displayed that L02 hepatocytes in the control group with normal ΔΨ*m* exhibited high fluorescence intensity for TMRM, while Cr(VI) treatment led to reduced TMRM dye and low fluorescence intensity, accompanying with the decrease of ATP content, mitochondria mass, and mtDNA copy number. Additionally, mitochondria appeared to be granulated (Figures 3(b)–3(e)). Furthermore, pretreatment with NAC partially rescued mitochondria depolarization, as demonstrated by the higher ratio of red/green fluorescence intensity with JC-1 probe in the NAC-treated cells compared with the cells treated with Cr(VI) alone (Figure 4(a)). Meanwhile, NAC pretreatment also partially inhibited the Cr(VI)-induced mitochondria dysfunction characterized by decreased mitochondria mass and mtDNA copy number as well as mitochondria granulation (Figures 4(b)–4(d)).

### 3.3. Cr(VI)-Induced Premature Senescence of L02 Hepatocytes Was Accompanied by Ca^2+^ Imbalance and NF-*κ*B Activation

**Figure 5:**
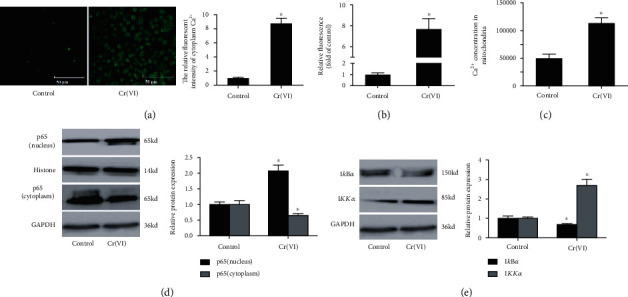
Cr(VI) caused intracellular Ca^2+^ overload and NF-*κ*B activation. The L02 hepatocytes were exposed to Cr(VI) for 4 weeks. (a) The mean fluorescence intensity intracellular Ca^2+^ concentration was determined by light microscopy (the micrograph of the cells, 200x), and ImageJ software was used to analyze the relative fluorescent intensity showed in bar graph; (b) spectrofluorometry was used to detect the change of Ca^2+^ concentration in cytoplasm. (c) Rhod-2 was used to assay the concentration of Ca^2+^ in mitochondria. (d) The protein levels of p65 (cytoplasm and nucleus) were determined by Western blot. (e) The expression of I*κ*B*α* and IKK*α* was detected by Western blot after Cr(VI) treatment. ImageJ software was used to analyze the relative levels of proteins normalized to the expression of GAPDH. All experiments were repeated at least 3 times and expressed as mean ± SD. ^∗^*p* < 0.05, compared with control. For the sake of clarity, the same control GAPDH was applied to compare with all experimentally relevant proteins with the same exposure time detected on the same SDS-PAGE gel unless otherwise stated.

Ca^2+^ has long been known to modulate cellular senescence, and mitochondria are regarded as a participant in Ca^2+^ signaling [29]. Thus, the intracellular and mitochondria Ca^2+^ levels were measured using the Fluo-3-AM and Rhod-2 fluorescent probe.  Figure 5(a) showed a striking increase in the Fluo-3-AM mean fluorescence using fluorescent microscopy in senescent L02 cells. The fluorescence of each group (L02 vs. senescent L02) was then quantitated by spectrofluorometry (the values in all cells), and the results indicated the elevated concentration of intracellular Ca^2+^. As expected, levels of Ca^2+^ in mitochondria were also upregulated in senescent L02 cells compared with the control group (Figure 5(b)) and were also upregulated in senescent L02 cells compared with the control group. Together, these results suggested that Ca^2+^ overload occurred in Cr(VI)-induced premature senescence of L02 hepatocytes. We also examined the relevant protein expression of the NF-*κ*B signaling pathway by Western blot for p65 (cytoplasm and nucleus), I*κ*B*α*, and IKK*α*. As demonstrated in Figure 5(c), the p65 protein in the nucleus was significantly increased accompanying with decreased protein levels of p65 in the cytoplasm. Further, we observed that I*κ*B*α*, the arrestin of p65, after Cr(VI) treatment was remarkably reduced, while there was an increase in IKK*α* (Figure 5(d)).

### 3.4. Ca^2+^ Overload and NF-*κ*B Activation in Senescent L02 Is Dependent on ROS Generation Induced by Cr(VI)

**Figure 6:**
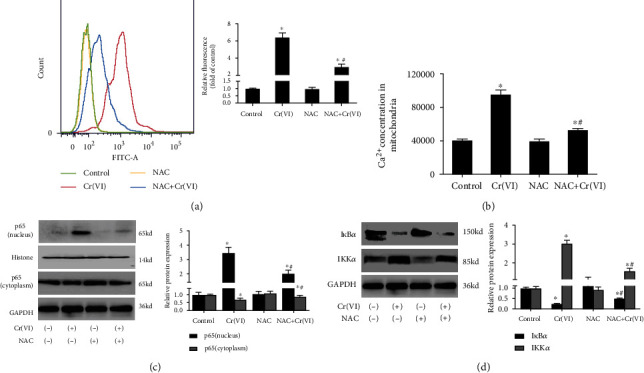
The intracellular Ca^2+^ overload and NF-*κ*B activation caused by Cr(VI) depended on ROS accumulation. The L02 cells were pretreated with NAC for 1 h prior to Cr(VI) treatment for 4 weeks. (a) The intracellular Ca^2+^ concentration was assayed by flow cytometry (histogram) and spectrofluorometry (bar graph). (b) The fluorescence of mitochondria Ca^2+^ was quantitated via spectrofluorometry. (c) The protein levels of p65 (cytoplasm and nucleus) and (d) I*κ*B*α* and IKK*α* were determined by Western blot. ImageJ software was used to analyze the relative levels of proteins normalized to the expression of GAPDH. All experiments were repeated at least 3 times and expressed as mean ± SD. ^∗^*p* < 0.05, compared with control; ^#^*p* < 0.05, compared with Cr(VI)-exposed group. For the sake of clarity, the same control GAPDH was applied to compare with all experimentally relevant proteins with the same exposure time detected on the same SDS-PAGE gel unless otherwise stated.

In our further study, we examined the connection between ROS, Ca^2+^ overload, and NF-*κ*B signaling pathway using antioxidant NAC pretreatment prior to Cr(VI) exposure. The quantitative analysis from flow cytometry and spectrofluorometry revealed increased intracellular Ca^2+^ concentration in senescent L02 which could be effectively restored by NAC pretreatment (Figure 6(a)). Accordingly, the mitochondria Ca^2+^ concentration analysis in Figure 6(b) found that the levels of mitochondrial Ca^2+^ of cells with NAC pretreated Cr(VI) exposure were significantly reduced, contrasting that in the Cr(VI)-along control group. These results suggested that the Ca^2+^ overload in Cr(VI)-induced senescent L02 was modulated by ROS accumulation. It has been proposed by our previous study that NF-*κ*B was a transcription factor that could be activated by ROS in Cr(VI)-induced cell apoptosis. We thus inferred that the activated NF-*κ*B signaling in Cr(VI)-induced senescent L02 may be mediated by ROS. In agreement with our hypothesis, results in Figures 6(c) and 6(d) indicated that the alteration of protein levels of NF-*κ*B signaling caused by Cr(VI) were significantly restored by NAC.

### 3.5. Cr(VI)-Induced NF-*κ*B Activation and Proinflammatory Cytokine Secretion in Senescent L02 Cells Were Mediated by Ca^2+^ Overload

**Figure 7:**
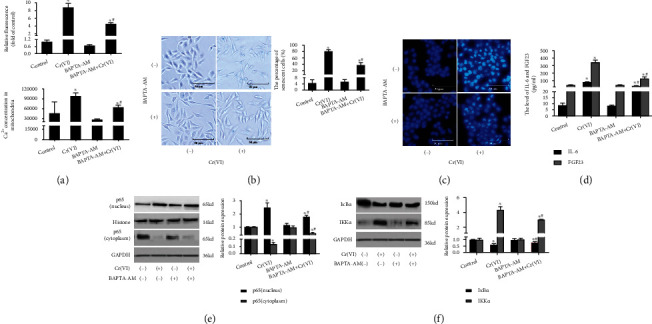
Ca^2+^ overload related to NF-*κ*B activation and proinflammatory cytokines secretion induced by Cr(VI). The L02 cells were pretreated with BAPTA-AM prior to Cr(VI) treatment for 4 weeks. (a) The cytoplasmic and mitochondrial Ca^2+^ concentration was determined by spectrofluorometry. (b) *β*-Galactosidase Staining Kit (200x) (the percentage of senescent cells was showed in bar graph) and (c) DAPI staining were used to detect SAHF (200x). (d) The secretion of IL-6 and FGF23 were assayed by ELISA kit. (e) The protein levels of p65 (cytoplasm and nucleus) and (f) I*κ*B*α* and IKK*α* were determined by Western blot. ImageJ software was used to analyze the relative levels of proteins normalized to the expression of GAPDH. All experiments were repeated at least 3 times and expressed as mean ± SD. ^∗^*p* < 0.05, compared with control; ^#^*p* < 0.05, compared with Cr(VI)-exposed group. For the sake of clarity, the same control GAPDH was applied to compare with all experimentally relevant proteins with the same exposure time detected on the same SDS-PAGE gel unless otherwise stated.

Emerging reports have suggested the key role of Ca^2+^ signaling in cellular senescence in the last decade [29], which makes us consider whether Ca^2+^ imbalance was involved in premature senescence, proinflammatory cytokines secretion, and NF-*κ*B activation caused by Cr(VI). L02 hepatocytes were pretreated with BAPTA-AM prior to Cr(VI) exposure before the determination of Ca^2+^. As seen in Figure 7(a), BAPTA-AM treatment alleviated Cr(VI)-induced intracellular and mitochondrial Ca^2+^ elevation to a certain extent. Results showed that the elevated SA-*β*-gal activity (Figure 7(b)) and SAHF (Figure 7(c)) under Cr(VI) exposure were effectively inhibited by BAPTA-AM pretreatment. Afterwards, results of ELISA assays also detected that treatment with BAPTA-AM resulted in obvious decrease of the levels of IL-6 and FGF23 (Figure 7(d)). Further, the related proteins of NF-*κ*B signaling were also assessed by Western blot. As shown in Figures 7(e) and 7(f), similar protective effects of BAPTA-AM were exhibited on p65, I*κ*B*α*, and IKK*α*. Taken together, it could be concluded that the premature senescence, secretion of IL-6 or FGF23, and NF-*κ*B activation of Cr(VI)-induced L02 hepatocytes were mediated by Ca^2+^ overload.

### 3.6. NF-*κ*B Participated in Cr(VI)-Induced Premature Senescence via Regulating Proinflammatory Cytokine Secretion

**Figure 8:**
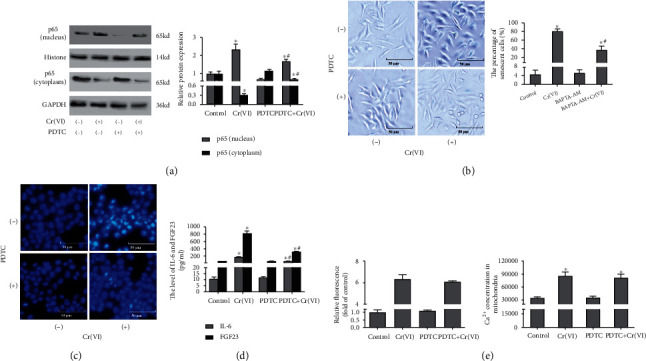
Active NF-*κ*B potentiates premature senescence without influencing intracellular Ca^2+^ concentration. PDTC was used to pretreat the L02 hepatocytes before Cr(VI) exposure for 4 weeks. (a) p65 protein (cytoplasm and nucleus) levels were detected by Western blot, and ImageJ software was used to analyze the relative levels of proteins normalized to the expression of GAPDH. (b) *β*-Galactosidase Staining Kit (200x) and (c) DAPI staining were used to detect SAHF (200x). (d) IL-6 and FGF23 secretion was analyzed by ELISA kit. (e) The fluorescence of intracellular and mitochondria Ca^2+^was quantitated via spectrofluorometry. All experiments were repeated at least 3 times and expressed as mean ± SD. ^∗^*p* < 0.05, compared with control; ^#^*p* < 0.05, compared with Cr(VI)-exposed group.

We next examined whether NF-*κ*B signaling involved in the chronic toxic effects in L02 hepatocytes was caused by Cr(VI). The inhibition efficiency of PDTC was assayed by Western blot analysis, and Figure 8(a) found that the altered p65 expression in the cytoplasm and nucleus attributed to Cr(VI) exposure was partially rescued by PDTC. Also, the increased SA-*β*-gal was obviously restored by PDTC pretreatment (Figure 8(b)), which could be further confirmed by DAPI staining on SAHF (Figure 8(c)). Next, ELISA assay validated that PDTC could robustly decrease the levels of IL-6 as well as FGF23 in L02 hepatocytes, supporting that NF-*κ*B is capable of regulating the secretion of IL-6 and FGF23 in Cr(VI)-induced senescent L02 hepatocytes (Figure 8(d)). Furthermore, we assessed the effects of PDTC on Ca^2+^ concentration (Figure 8(e)). Interestingly, our data showed that the treatment with 50 *μ*M PDTC did not prevent the Cr(VI)-induced intracellular and mitochondrial Ca^2+^ overload. Collectively, we concluded that Cr(VI) could induce premature senescence of L02 hepatocytes through upregulating NF-*κ*B signaling via secretion of IL-6 and FGF23.

## 4. Discussion

The role of cellular senescence in the development and progression of age-related diseases has been extensively explored in recent years. There is mounting evidence suggesting that aging promoted hyperplastic pathologies, the deadly of which is cancer [30]. In addition, a study indicated that obesity-induced gut microbial metabolite could promote liver cancer through senescence. There are three primary cellular senescence identified to date. Among them, stress-induced premature senescence may be the common cell fate due to multiple stressors that include, but are not limited to, DNA damage. Previous studies indicated that Cr(VI) could cause severe DNA damage [31]. It is generally believed that intrahepatic cell senescence is closely related to the pathogenesis of liver diseases, such as liver fibrosis and cirrhosis [32]. However, the stress-induced premature senescence in liver injury is still poorly understood. Though we previously demonstrated that Cr(VI) exerted the toxic effects of inducing premature senescence on L02 hepatocytes via the p53 signaling pathway [10], the underlying mechanism are not fully elucidated. Cr(VI), a highly toxic heavy metal, is broadly dispersed in the environment, which has various adverse effects on humans upon its exposure via inhalation, ingestion, and skin absorption especially pulmonary and liver injury [33, 34]. There are series of studies on the role and underlying modulatory mechanisms of Cr(VI)-induced acute cytotoxicity in liver injury to date [13, 35, 36], but less attention has hitherto been drawn to the chronic cytotoxicity such as premature senescence. This present study confirmed that a chronic Cr(VI) exposure could lead to premature senescence of hepatocytes, as highlighted by some common features with cells in natural aging including activation of SA-*β*-gal, increased H3K9me3 markers reflecting the formation of SAHF, and decreased proliferation profiles as well as G1 phase arrest. Additionally, other hallmarks of cellular senescence including p53/p21^WAF1/CIP1^siganling (p53, p21, and MDM2) and some indicators such as CTGF, SMP30, and Cav-1 were detected to confirm “true” senescence occurred. p53, the central regulator of cellular senescence, is mainly inducing p21 that is the inhibitor of cyclin-dependent kinases leading to G0/G1 phase arrest. Both MDM2 and Cav-1 play key roles in the activity of p53/p21^WAF1/CIP1^. MDM2 promotes the ubiquitinated degradation of p53, while Cav-1 could function as the inducer of p53 [37]. CTGF and SMP30 are also the useful marker for the identification of senescence [38]. These factors are significantly changed after Cr(VI) exposure.

Evidence suggests that persistent low-grade inflammation is the hallmark of senescence [38]. IL-6 is a critical inflammatory factor that has been displayed to reinforce the senescence program in an autocrine manner and to promote senescence induction in a paracrine mode [39]. Further, the role of FGF23 as an inflammatory facilitator to induce upregulation and secretion of IL-6 has also been recently studied. FGF23, a predominantly bone-derived hormone acting on the phosphate and vitamin D metabolism, requires *α*-klotho (an antiaging genes) as a coreceptor for its biological functions [40, 41]. Interestingly, increased FGF23 levels in connection with low *α*-klotho are believed to be correlated with aging [41]. In line with this, we also found that the protein levels of IL-6 and FGF23 were significantly increased in Cr(VI)-induced senescent hepatocytes.

Moreover, there is an evident correlation between inflammatory response and oxidative stress [42]. Results from in vitro and in vivo studies suggested that formation of ROS is able to function as signaling molecules under physiological conditions [43]. However, under pathological conditions, aberrant accumulation of intracellular ROS disturbs the redox homeostasis and subsequently leads to serious cell damage. There are already evidences that overproduction of ROS not only regulates Cr(VI)-induced acute cytotoxicity, for instance cell cycle arrest and apoptosis [44], but also is associated with Cr(VI)-induced chronic cytotoxicity, such as malignant transformation. Our present study showed that Cr(VI)-induced premature senescence was driven by excessive ROS, and antioxidant NAC pretreatment could partially rescue premature senescence and secretion of IL-6 and FGF23 caused by Cr(VI). Although NADPH oxidases, xanthine oxidase, the mitochondrial respiratory chain, lipoxygenases, and nitric oxide synthases are all thought to be source of ROS generation, the mitochondria have been regarded as the major supply of ROS in hepatocytes [45]. Excessive ROS formation causes electron transport chain dysfunction and disrupts the regulation of energy; accompanying with lipid peroxidation in the mitochondria membrane, decreased ΔΨ*m*, and abnormal mitochondrial morphology further contributes to the accumulation of ROS, leading to a vicious circle between ROS and mitochondria [46]. Herein, we have demonstrated experimental evidence that ROS derived from dysfunctional mitochondria with the mitochondrial depolarization is a critical upstream regulator of Cr(VI)-triggered premature senescence of hepatocytes.

It has reported that the initial response to intracellular ROS accumulation is the elevated Ca^2+^ influx, further regulated both the activity and the expression of several pivotal proteins involved in Ca^2+^ signaling, and in some cases, Ca^2+^ is required in many ROS-related cellular responses, which well demonstrated the interaction between ROS signaling mitochondria origin and Ca^2+^ homeostasis [47]. Indeed, Ca^2+^ signaling has emerged as a key player exploited by cells to tune their activity in accordance with biological functions. The different components, such as channels, pumps, antiporters, and Ca^2+^-binding proteins, interdependently cooperate to retain intracellular Ca^2+^ homeostasis. Under the resting condition, cytosolic Ca^2+^ concentration is maintained around 100 nM, while cytosolic Ca^2+^ concentration may rise up to 1~3 *μ*M when induced by different stimuli [14]. It is reported that intracellular Ca^2+^ obviously increased when the hepatocytes were exposed to Cr(VI), but the Cr(VI)-induced hepatotoxicity could be inhibited by removing media Ca^2+^, which suggests that the Ca^2+^ transporter is involve in Ca^2+^ accumulation caused by Cr(VI) contributing its cytotoxicity [48]. Our previous data revealed that the stromal interaction molecule 1 could be the key mediators of Ca^2+^ influx in L02 hepatocytes incubated with Cr(VI), which is thought to contribute to the elevated intracellular Ca^2+^ concentration [15]. The disordered intracellular Ca^2+^ homeostasis in hepatocytes with combined exposure to cadmium and BDE209 [14] further indicated that imbalanced Ca^2+^ homeostasis may act as a pivotal regulator for acute hepatotoxicity induced by xenobiotics. Significant attention has been also drawn on the role of Ca^2+^ homeostasis in senescence. Nevertheless, the involvement of abnormal Ca^2+^ homeostasis in the premature cellular senescence of hepatocytes remains somewhat controversial. The common notion is that there is an increase in the intracellular Ca^2+^ during senescence progression. It has been reported that a higher Ca^2+^ concentration in replicative senescent fibroblasts was noted compared to that in nonsenescent cells [49]. Another research demonstrated that rotenone-induced premature senescence in human neuroblastoma cells was accompanied with cytosolic Ca^2+^ rise [50]. Although there is a growing body of evidence that has revealed the involvement of disturbed Ca^2+^ homeostasis in cellular senescence and Cr(VI)-induced acute hepatotoxicity, the role of the elevated Ca^2+^ in Cr(VI)-induced premature senescence has not been investigated and remains controversial. We further observed the relationship between Ca^2+^ imbalance and Cr(VI)-induced premature senescence of hepatocytes. Consistent with the abovementioned studies, we also revealed an increase of Ca^2+^ levels of cytoplasm and mitochondria in Cr(VI)-exposed L02 hepatocytes, suggesting that Ca^2+^ regulated by ROS is involved in premature senescence of hepatocytes. In favor of this hypothesis, BAPTA-AM partially restored premature senescence in L02 hepatocytes, as detected by both SA-*β*-gal staining and partially decreased secretion of IL-6 and FGF23.

In addition, NF-*κ*B transcription factors are well-known regulators of senescence. According to our data, inhibition of NF-*κ*B transcription factors by PDTC was sufficient to prevent premature senescence induced by Cr(VI), which corroborates previous findings reported in other senescence contexts [26]. Of relevance is that activation of NF-*κ*B signaling was profoundly associated with Cr(VI)-triggered increase in the intracellular Ca^2+^ levels of L02 hepatocytes [15]. A recent study also stated that NF-*κ*B signaling was activated by elevated intracellular Ca^2+^ [51]. Our results were correlated well with those findings, with no upstream effect of NF-*κ*B on Ca^2+^ concentration being observed. Further research efforts are welcomed to analyze detailed correlations between the intracellular Ca^2+^ concentration and NF-*κ*B.

**Figure 9:**
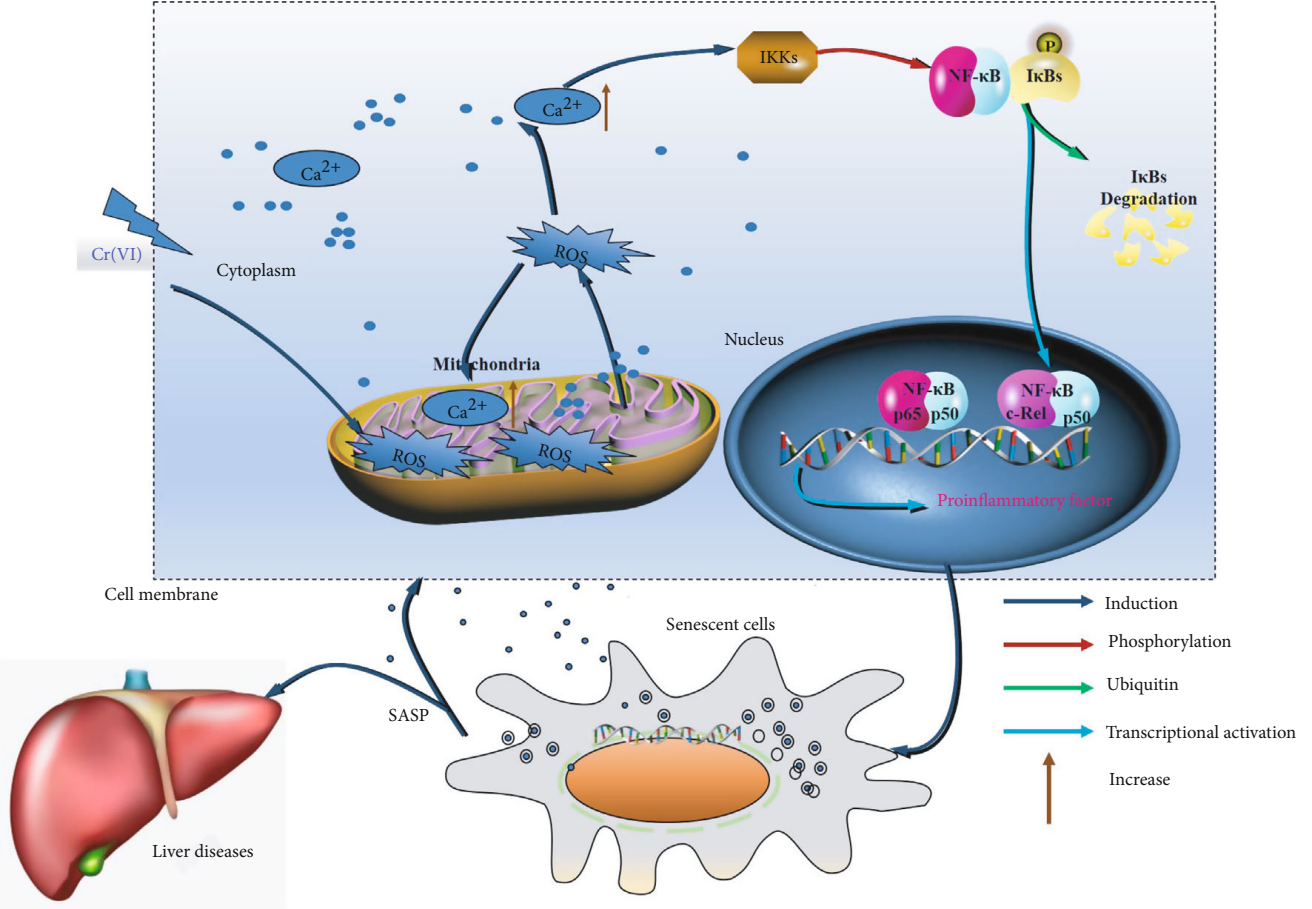
A schematic model. In Cr(VI)-exposed L02 hepatocytes, dysfunction of mitochondria made accumulation of the intracellular ROS contributing to subsequent Ca^2+^overload in cytoplasm/mitochondria and activation of NF-*κ*B signaling. In addition, NF-*κ*B, a transcription factor of inflammatory factors [52], is activated by Ca^2+^overload and then elevates the expression and secretion of inflammatory factors, inducing premature cellular senescence. Furthermore, SASP may lead to senescence-related inflammation, metabolic dysregulation, stem cell dysfunction, aging phenotypes, and liver diseases [53].

In conclusion, our study investigated the underlying mechanisms of Cr(VI)-induced premature senescence in L02 hepatocytes (as depicted in Figure 9). In our data, chronic low-dose Cr(VI) exposure could induce premature cellular senescence upon intracellular ROS accumulation that could positively regulate the intracellular concentration of Ca^2+^. In addition, Ca^2+^ directly activates NF-*κ*B signaling and further induces the expression of IL-6 and FGF23. To the best of our knowledge, our data proposes for the first time the role of ROS-Ca^2+^-NF-*κ*B signaling pathway in Cr(VI)-induced premature senescence, which may provide new targets for molecular intervention and treatment for aging-related chronic disorders.

## Data Availability

Data and materials are included in the manuscript.

## Supplementary Materials

Supplementary Materials There was no obvious cell apoptosis in the control and Cr(VI)-exposed group via flow cytometry and TUNEL analysis (Supplementary Fig. S1), suggesting that senescence was not related to cell apoptosis. The protein levels of age-related biomarkers including p53, MDM2, p21, CTGF, SMP30, and Cav-1 were detected by Western blot, which were significantly changed (Supplementary Figure S2).

## References

[1] Paluvai H., Di Giorgio E., Brancolini C. (2020). The histone code of senescence. *Cells*.

[2] Kumar S., Suman S., Fornace A. J., Datta K. (2019). Intestinal stem cells acquire premature senescence and senescence associated secretory phenotype concurrent with persistent DNA damage after heavy ion radiation in mice. *Aging (Albany NY)*.

[3] Kida Y., Goligorsky M. S. (2016). Sirtuins, cell senescence, and vascular aging. *The Canadian Journal of Cardiology*.

[4] Zhang Y., Zhang Y., Xiao Y., Zhong C., Xiao F. (2019). Expression of clusterin suppresses Cr(VI)-induced premature senescence through activation of PI3K/AKT pathway. *Ecotoxicology and Environmental Safety*.

[5] Seki E., Brenner D. A. (2015). Recent advancement of molecular mechanisms of liver fibrosis. *Journal of Hepato-Biliary-Pancreatic Sciences*.

[6] Hakeem A., Reza S., Moinuddin S. (2007). Cirrhosis in Werner's syndrome: an unusual presentation of premature aging. *Medical Science Monitor*.

[7] Oubaha M., Miloudi K., Dejda A. (2016). Senescence-associated secretory phenotype contributes to pathological angiogenesis in retinopathy. *Science Translational Medicine*.

[8] Cardoso A. L., Fernandes A., Aguilar-Pimentel J. A. (2018). Towards frailty biomarkers: candidates from genes and pathways regulated in aging and age-related diseases. *Ageing Research Reviews*.

[9] Niemann J., Johne C., Schröder S. (2017). An mtDNA mutation accelerates liver aging by interfering with the ROS response and mitochondrial life cycle. *Free Radical Biology & Medicine*.

[10] Zhang Y., Zhang Y., Zhong C., Xiao F. (2016). Cr(VI) induces premature senescence through ROS-mediated p53 pathway in L-02 hepatocytes. *Scientific Reports*.

[11] Chen Q. Y., Murphy A., Sun H., Costa M. (2019). Molecular and epigenetic mechanisms of Cr(VI)-induced carcinogenesis. *Toxicology and Applied Pharmacology*.

[12] Linos A., Petralias A., Christophi C. A. (2011). Oral ingestion of hexavalent chromium through drinking water and cancer mortality in an industrial area of Greece--an ecological study. *Environmental Health*.

[13] Hempel N., Trebak M. (2017). Crosstalk between calcium and reactive oxygen species signaling in cancer. *Cell Calcium*.

[14] Wang L., Zheng M., Zhang S., Zhao C., Kang W., Wang K. (2019). Roles of mtDNA damage and disordered Ca^2+^ homeostasis in the joint toxicities of cadmium and BDE209. *Ecotoxicology and Environmental Safety*.

[15] Yi X., Zhang Y., Zhong C., Zhong X., Xiao F. (2016). The role of STIM1 in the Cr(vi)-induced [Ca2+]iincrease and cell injury in L-02 hepatocytes. *Metallomics*.

[16] Borodkina A. V., Shatrova A. N., Deryabin P. I. (2016). Calcium alterations signal either to senescence or to autophagy induction in stem cells upon oxidative stress. *Aging (Albany NY)*.

[17] Calvo-Rodriguez M., Hernando-Pérez E., López-Vázquez S., Núñez J., Villalobos C., Núñez L. (2020). Remodeling of intracellular Ca^2+^ homeostasis in rat hippocampal neurons aged in vitro. *International journal of molecular sciences*.

[18] Madreiter-Sokolowski C. T., Waldeck-Weiermair M., Bourguignon M. P. (2019). Enhanced inter-compartmental Ca^2+^ flux modulates mitochondrial metabolism and apoptotic threshold during aging. *Redox biology*.

[19] Herraiz-Martínez A., Álvarez-García J., Llach A. (2015). Ageing is associated with deterioration of calcium homeostasis in isolated human right atrial myocytes. *Cardiovascular Research*.

[20] Franca A., Filho A., Guerra M. T. (2019). Effects of endotoxin on type 3 inositol 1,4,5-trisphosphate receptor in human cholangiocytes. *Hepatology*.

[21] Choi M. C., Jo J., Park J., Kang H. K., Park Y. (2019). NF-B signaling pathways in osteoarthritic cartilage destruction. *Cell*.

[22] Yang Q., Han B., Li S. (2022). The link between deacetylation and hepatotoxicity induced by exposure to hexavalent chromium. *Journal of advanced research*.

[23] Cartwright T., Perkins N. D., Wilson C. L. (2016). NFKB1: a suppressor of inflammation, ageing and cancer. *The FEBS Journal*.

[24] Tian Y., Li H., Qiu T. (2019). Loss of PTEN induces lung fibrosis via alveolar epithelial cell senescence depending on NF-*κ*B activation. *Aging Cell*.

[25] Liang Y., Liang N., Ma Y., Tang S., Ye S., Xiao F. (2021). Role of clusterin/NF-*κ*B in the secretion of senescence-associated secretory phenotype in Cr(VI)-induced premature senescent L-02 hepatocytes. *Ecotoxicology and Environmental Safety*.

[26] Fão L., Mota S. I., Rego A. C. (2019). Shaping the Nrf2-ARE-related pathways in Alzheimer's and Parkinson's diseases. *Ageing Research Reviews*.

[27] Pi H., Xu S., Zhang L. (2013). Dynamin 1-like-dependent mitochondrial fission initiates overactive mitophagy in the hepatotoxicity of cadmium. *Autophagy*.

[28] Saxena S., Vekaria H., Sullivan P. G., Seifert A. W. (2019). Connective tissue fibroblasts from highly regenerative mammals are refractory to ROS-induced cellular senescence. *Nature Communications*.

[29] Janikiewicz J., Szymański J., Malinska D. (2018). Mitochondria-associated membranes in aging and senescence: structure, function, and dynamics. *Cell Death & Disease*.

[30] Calcinotto A., Kohli J., Zagato E., Pellegrini L., Demaria M., Alimonti A. (2019). Cellular senescence: aging, cancer, and injury. *Physiological Reviews*.

[31] Ren X., Xia B., Chen Z. (2019). Short-term and long-term exposure to hexavalent chromium alters 53BP1 via H3K18ac and H3K27ac. *Chemosphere*.

[32] Luo X., Jiang X., Li J. (2019). Insulin-like growth factor-1 attenuates oxidative stress-induced hepatocyte premature senescence in liver fibrogenesis via regulating nuclear p53-progerin interaction. *Cell Death & Disease*.

[33] Zhang T. G., Zhao Y. L., Li L., Zhou D. H. (2020). Antagonistic effects of nano-selenium on broilers hepatic injury induced by Cr((VI)) poisoning in AMPK pathway. *Environmental Science and Pollution Research International*.

[34] Gao Z., Mei J., Yan X. (2020). Cr (VI) induced mitophagy via the interaction of HMGA2 and PARK2. *Toxicology Letters*.

[35] Zhang Y., Ma Y., Xiao Y., Lu C., Xiao F. (2020). Drp1-dependent mitochondrial fission contributes to Cr(VI)-induced mitophagy and hepatotoxicity. *Ecotoxicology and Environmental Safety*.

[36] Yang Q., Han B., Xue J. (2020). Hexavalent chromium induces mitochondrial dynamics disorder in rat liver by inhibiting AMPK/PGC-1*α* signaling pathway. *Environmental Pollution*.

[37] Jiang C., Liu G., Luckhardt T. (2017). Serpine 1 induces alveolar type II cell senescence through activating p53-p21-Rb pathway in fibrotic lung disease. *Aging Cell*.

[38] Hernandez-Segura A., Nehme J., Demaria M. (2018). Hallmarks of cellular senescence. *Trends in cell biology*.

[39] Ortiz-Montero P., Londoño-Vallejo A., Vernot J. P. (2017). Senescence-associated IL-6 and IL-8 cytokines induce a self- and cross-reinforced senescence/inflammatory milieu strengthening tumorigenic capabilities in the MCF-7 breast cancer cell line. *Cell Communication and Signaling: CCS*.

[40] Krick S., Helton E. S., Hutcheson S. B. (2018). FGF23 induction of O-linked N-Acetylglucosamine regulates IL-6 secretion in human bronchial epithelial cells. *Front Endocrinol (Lausanne)*.

[41] Chen G., Liu Y., Goetz R. (2018). *α*-Klotho is a non-enzymatic molecular scaffold for FGF23 hormone signalling. *Nature*.

[42] Lin Y., Jiang M., Chen W., Zhao T., Wei Y. (2019). Cancer and ER stress: mutual crosstalk between autophagy, oxidative stress and inflammatory response. *Biomedicine & Pharmacotherapy*.

[43] Zhong H., Song R., Pang Q. (2018). Propofol inhibits parthanatos via ROS-ER-calcium-mitochondria signal pathway in vivo and vitro. *Cell Death & Disease*.

[44] Zhang Y., Ma Y., Liang N., Liang Y., Lu C., Xiao F. (2019). Blockage of ROS-ERK-DLP1 signaling and mitochondrial fission alleviates Cr(VI)-induced mitochondrial dysfunction in L02 hepatocytes. *Ecotoxicology and Environmental Safety*.

[45] Stefanatos R., Sanz A. (2018). The role of mitochondrial ROS in the aging brain. *FEBS Letters*.

[46] Lv Y., Jiang H., Li S. (2020). Sulforaphane prevents chromium-induced lung injury in rats via activation of the Akt/GSK-3*β*/Fyn pathway. *Environmental pollution (Barking, Essex)*.

[47] Yan Y., Wei C. L., Zhang W. R., Cheng H. P., Liu J. (2006). Cross-talk between calcium and reactive oxygen species signaling. *Acta Pharmacologica Sinica*.

[48] Yi X., Xiao F., Zhong X., Duan Y., Liu K., Zhong C. (2017). A Ca^2+^ chelator ameliorates chromium (VI)-induced hepatocyte L-02 injury via down-regulation of voltage-Dependent anion channel 1 (VDAC1) expression. *Environmental Toxicology and Pharmacology*.

[49] Lee T. Y., Huang L. J., Dong H. P., Tohru Y., Liu B. H., Yang R. C. (2020). Impairment of mitochondrial unfolded protein response contribute to resistance declination of H2O2‐induced injury in senescent MRC-5 cell model. *The Kaohsiung Journal of Medical Sciences*.

[50] Ricke K. M., Paß T., Kimoloi S. (2020). Mitochondrial dysfunction combined with high calcium load leads to impaired antioxidant defense underlying the selective loss of nigral dopaminergic neurons. *The Journal of Neuroscience*.

[51] Gong W. X., Zhou Y. Z., Qin X. M., Du G. H. (2019). Involvement of mitochondrial apoptotic pathway and MAPKs/NF-*κ* B inflammatory pathway in the neuroprotective effect of atractylenolide III in corticosterone-induced PC12 cells. *Chinese Journal of Natural Medicines*.

[52] Lawrence T. (2009). The nuclear factor NF-B pathway in inflammation. *Cold Spring Harbor Perspectives in Biology*.

[53] Huda N., Liu G., Hong H., Yan S., Khambu B., Yin X. M. (2019). Hepatic senescence, the good and the bad. *World Journal of Gastroenterology*.

