# Supplementary figures and images for: Regulation of Cr(VI)-Induced Premature Senescence in L02 Hepatocytes by ROS-Ca2+-NF-κB Signaling
Source: Oxid Med Cell Longev. 2022 Feb 18;2022:7295224. doi: 10.1155/2022/7295224 (PMC8881123; doi:10.1155/2022/7295224)

**(A)**

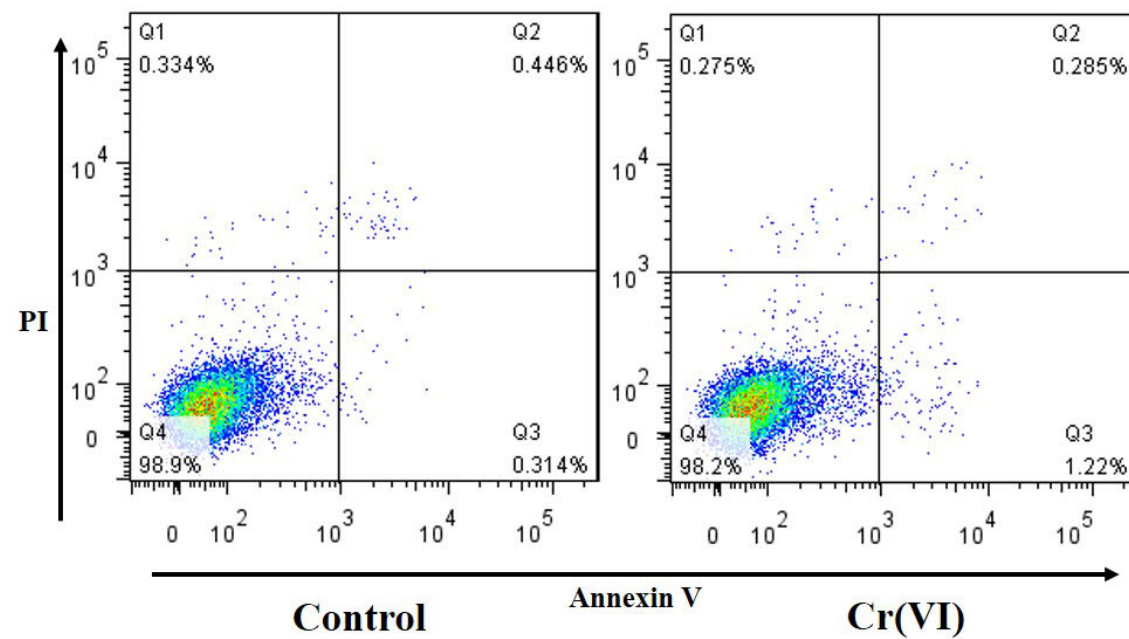

**(B)**

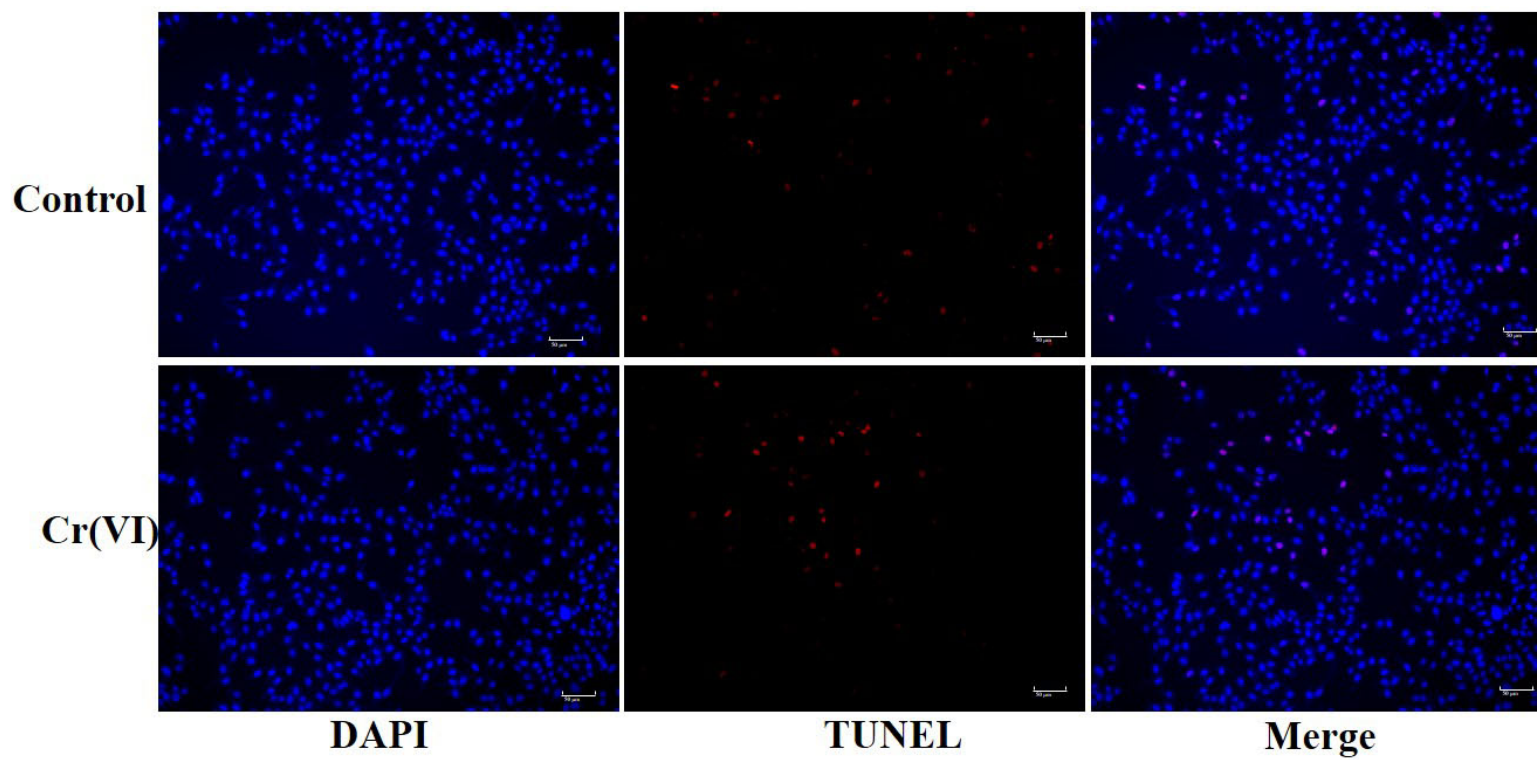

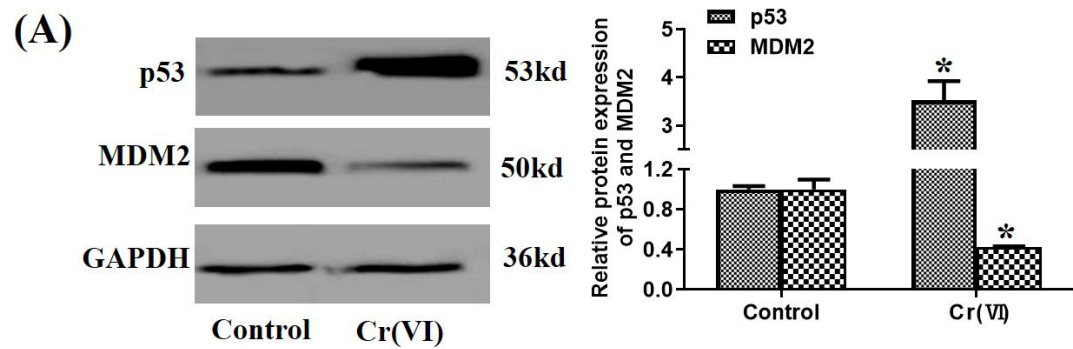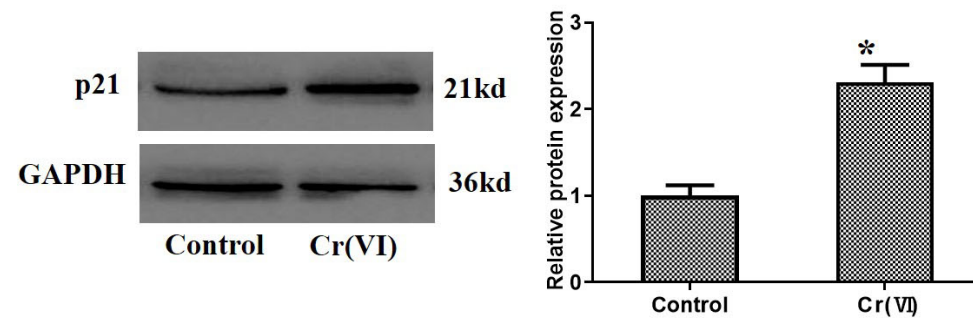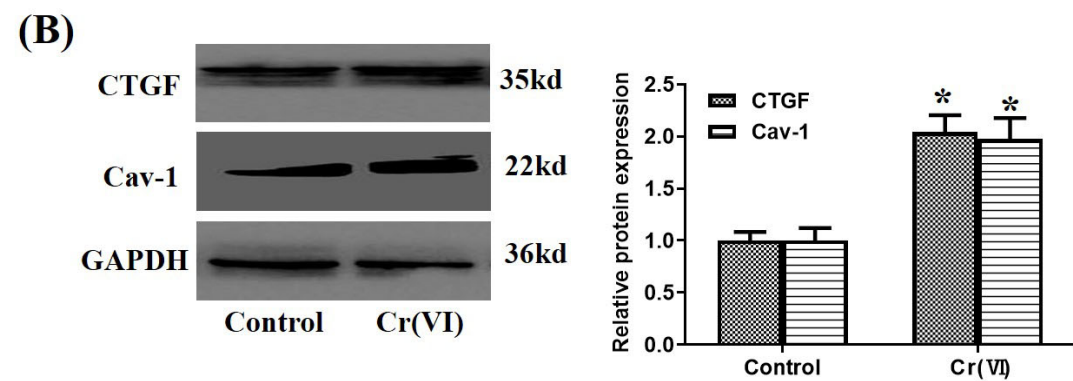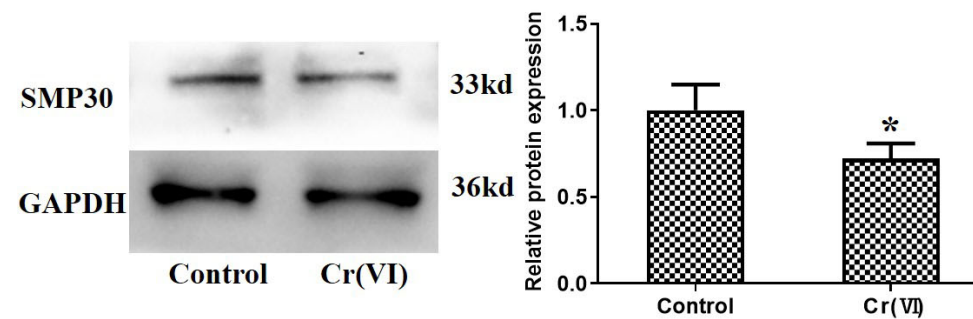

Supplement: Supplementary Materials — There was no obvious cell apoptosis in the control and Cr(VI)-exposed group via flow cytometry and TUNEL analysis (Supplementary Fig. S1), suggesting that senescence was not related to cell apoptosis. The protein levels of age-related biomarkers including p53, MDM2, p21, CTGF, SMP30, and Cav-1 were detected by Western blot, which were significantly changed (Supplementary Figure S2). [file 7295224.f1.pdf]
